# Exploring Current Medical Learning: A Comprehensive Analysis of Internet Usage Patterns and Educational Impact Among Medical Students

**DOI:** 10.7759/cureus.81003

**Published:** 2025-03-22

**Authors:** Manisha Pandey, Anita Rani, Prashant K Bajpai, Jyoti Chopra

**Affiliations:** 1 Anatomy, Dr. Ram Manohar Lohia Institute of Medical Sciences, Lucknow, IND; 2 Anatomy, King George's Medical University, Lucknow, IND; 3 Community Medicine and Public Health, King George's Medical University, Lucknow, IND

**Keywords:** digital learning, first-year students, internet use, medical education, online resources, study habits

## Abstract

Introduction

In recent years, the integration of technology into medical education has profoundly altered conventional learning environments. The extensive use of the internet, digital resources, and online platforms has facilitated rapid access to substantial information for students, thus augmenting their capacity to engage with course content in more dynamic and engaging ways. Nonetheless, along with these benefits, challenges have arisen, particularly in students' management and regulation of their internet use for academic purposes. This study aimed to analyze the factors affecting internet use for study-related purposes and to identify the strengths and challenges in regulating its academic usage.

Methods

A cross-sectional study was undertaken among 144 first-year MBBS students at a government medical college in Lucknow, India. This study specifically included participants from the first year of the MBBS program, aged between 18 and 25 years. Students who did not provide explicit consent to participate or were absent on the designated day of data collection were excluded. Data were gathered using a predesigned, semi-structured questionnaire addressing demographics, internet usage patterns, preferences, and perceptions. Statistical analysis was conducted using SPSS version 24.0.

Results

While 108 (75%) of students preferred textbooks, 99 (68.8%) used the internet for study-related activities. Google Chrome (N=79, 79.8%) was the preferred search engine, and Wikipedia (N=63, 63.6%) was the most common database. Mobile internet access (N=59, 60%) and Android devices (N=86, 86.9%) were predominant. Most students (N=76, 76.8%) perceived the content reliability as moderate (based on students' subjective perceptions), with only 33 (33.3%) fully satisfied with internet services. Slow internet speed (based on students' subjective perceptions) was the primary challenge reported by 63 (63.6%) students.

Conclusions

The study highlights the evolving digital learning landscape in medical education, with a mix of traditional and online resources being utilized. While the internet offers benefits like unlimited access to study materials, challenges such as slow speeds persist. Future research should focus on enhancing content reliability and user satisfaction and exploring the impact of emerging technologies.

## Introduction

Technology has become a transformative force in the ever-evolving landscape of education, shaping students’ and educators’ learning experiences. This technological paradigm shift facilitates ease in daily life and serves as a conduit to connect individuals with the latest developments across diverse fields, such as communication, research, and entertainment. Notably, Web technologies have brought about a profound revolution, particularly in education, offering unprecedented opportunities for quality learning at an affordable cost [1]. As of the onset of 2020, an estimated 700 million individuals were utilizing the Internet, with projections indicating a surge to 974 million by 2025. This exponential growth holds immense implications for national economies, and India, in particular, secured its second position globally in the online marketplace in 2019 [2]. The accessibility of various resource materials, including e-books, audio, images, and educational videos, has become effortlessly attainable, fostering a gradual shift towards utilizing the internet as an educational tool [3]. The constant evolution of advancements has necessitated health professionals to stay abreast of the latest developments in the medical domain. Affordable and uninterrupted Internet facilities are pivotal for facilitating this ongoing learning process. The Internet has become a vast repository of medical information, with numerous Internet-based platforms offering medical students opportunities to enhance their competencies at their own pace [4, 5]. These platforms not only serve as valuable resources for learning but also contribute to the development of research acumen among students [6]. The availability of large amounts of information helps students explore their areas of interest and gain in-depth knowledge, which may lead to developing research understanding [7-9]. Termed Generation Z (Gen Zers), the current generation has unfettered access to the Internet, and their learning preferences are significantly influenced by technology. Pedagogical approaches involving videos, visuals, graphic organizers, 3D animations, gaming, quizzes, and class blogs have proven particularly appealing, enhancing the overall learning experience. Despite the transformative potential, there remains a scarcity of studies investigating the role of web technologies in academic settings. Medical students' perception of Internet usage for study-related endeavors is an underexplored area [10, 11]. According to a Rathod PG et al. (2023) study, the main ways that smartphones affect academic performance are enhanced learning skills (60.91%), prompt assignment completion (40%), more discussion participation (34.55%), and the development of critical and creative thinking (29.09%) [12]. In alignment with the evolving landscape of education and the recently launched Digital India campaign under the 2020 National Education Policy, the objective of the present study is to analyze the factors affecting Internet use for study-related purposes and identify the strengths and challenges regulating its academic usage. This exploration is imperative for understanding the dynamic interplay between technology and medical education, providing valuable insights for educators, policymakers, and medical professionals.

## Materials and methods

Study design and study population

This cross-sectional study was designed and executed among first-year MBBS students at a prominent Government Medical College in Lucknow. First-year MBBS students were chosen because they are in the transition phase from traditional learning methods to digital learning. The investigation commenced six months after the students’ admission to gain insights into their evolving utilization of digital resources for educational purposes.

Study tool

To comprehensively capture the diverse aspects of internet usage, a predesigned, pretested, and semi-structured questionnaire was employed as the primary study tool. This instrument aimed to delve into the multifaceted dimensions of internet-related behaviors, preferences, and perceptions among the participating students.

Inclusion criteria

The study included participants from the first phase of the MBBS program, specifically targeting individuals aged between 18 and 25 years. This age range was chosen to focus on the unique characteristics and preferences of young adult learners in the early stages of their medical education.

Exclusion criteria

Stringent exclusion criteria were applied to ensure the accuracy and reliability of the data. Students who did not provide explicit consent to participate in the study and those who were absent on the designated day of data collection were excluded.

Sample size and sampling method

The initial pool comprised 250 first-phase MBBS students. The study included all eligible students within the institution, making it a complete enumeration rather than a random sample. Applying a rigorous inclusion and exclusion criteria process, the study sample was refined to 144 participants using a census method to comprehensively represent the targeted population.

Methodology

Before distributing the questionnaire, an interactive orientation session was conducted with all the Phase I students. This session provided an opportunity to elucidate the nature of the study, its objectives, and expected outcomes. Informed consent was obtained to establish rapport with the participants, underlining the importance of candid and authentic responses. The questionnaire covered a spectrum of demographic information, including age, sex, residential area, education medium, and type of school attended. Additionally, participants were queried about their preferences related to search engines, devices, sources of internet access, and databases utilized in their study-related activities. Beyond the instrumental aspects, the questionnaire explored participants' perceptions regarding the reliability of information, satisfaction levels with internet services, perceived benefits, challenges encountered in study-related activities, and preferred sources for learning. Participation was voluntary, and efforts were made to ensure representativeness by including students from diverse backgrounds. Non-response bias was minimized by conducting multiple follow-ups.

Ethical aspects

The study adhered to ethical standards, rigorously maintained confidentiality, and each participant provided informed consent, ensuring their rights and privacy were respected throughout the study.

Data entry and statistical analysis

To ensure robust analysis, the collected data were meticulously entered using SPSS (version 24.0; IBM Corp., USA). Results are expressed as proportions, and the chi-square test was used to assess statistical significance. A p-value <0.05 was considered as the threshold for determining statistical significance. This analytical approach aimed to derive meaningful insights from the dataset, contributing to scholarly discourse on the evolving intersection of medical education and digital technology.

## Results

When participants were asked about their preferred study materials, a significant majority, 108 students (75.0%), chose textbooks. Only 11 students (7.6%) preferred solely internet-based content, while another 11 students (7.6%) used a combination of both textbooks and the internet (Table 1). Of the 144 respondents, 99 students (68.8%) used the internet for study-related activities. Among those who used the internet for study-related activities (99 students), a predominant proportion were male (62 students, 43.1%), residents of urban areas (64 students, 44.4%), and had completed their 12th standard education in English-medium public or convent schools (77 students, 53.5%). However, no statistically significant differences were observed based on gender, residence, type of school, or medium of instruction (Table 2).

**Table 1:** Material preferences for study among medical students (n=144).

Preferred Source	Number	Percentage
Books	108	75.0
Internet	11	7.6
Both	11	7.6
No Response	14	9.8

**Table 2:** Association of internet utilization with bio-social characteristics in study subjects. P-value <0.05 was considered significant.

Bio-social Characteristics		Use of Internet		P-value
		Yes	No	
Gender	Male	62 (43.1)	31 (21.5)	0.466
	Female	37 (25.7)	14 (9.7)	
Residence	Rural	35 (24.3)	17 (11.8)	0.779
	Urban	64 (44.4)	28 (19.4)	
Type of school from which they passed 12th standard	Government	38 (26.4)	18 (12.5)	0.854
	Public & Convent	61 (42.4)	27 (18.8)	
Type of medium of school from which they passed 12th standard	English	77 (53.5)	36 (25.0)	0.764
	Hindi	22 (15.3)	9 (6.2)	

Google Chrome emerged as the preferred search engine, with 79 students (79.8%) using it for internet searches (Table 3). The most commonly used database for learning was Wikipedia, with a usage rate of 63 students (63.6%). Interestingly, when students were allowed to select multiple responses, 84 students (58.3%) still favored Wikipedia. In contrast, only 2 students (2.0%) reported using PubMed. A total of 59 students (59.6%) opted for mobile internet access. The Android operating system dominated, being used by 86 students (86.9%) during internet searches (Table 4).

**Table 3:** Search engine utilization patterns in internet-engaged students (n=99, i.e., students who used the internet).

	Number	Percentage (%)
Safari/Bing/Yahoo/ask.com/Firefox/Internet explorer	12	12.1
Google Chrome	79	79.8
Do not know	8	8.1

**Table 4:** Utilization metrics: databases, internet sources, and operating systems in study subjects.

Variable	Number (Single response)	Percentage (Single response)	Number (Multiple response)	Percentage (Multiple response)
ClinicalKey	16	16.20%	22	15.30%
PubMed	2	2.00%	2	1.40%
Wikipedia	63	63.60%	84	58.30%
Google Scholar	5	5.10%	7	4.90%
All	1	1.00%	17	11.80%
No Response	12	12.10%	12	8.30%
Total	99	100.00%	144	100.00%
Dongle/Datacard	3	3.00%	9	6.00%
Wi-Fi	26	26.30%	35	23.50%
Mobile Internet	59	59.60%	92	61.80%
Landline	2	2.00%	4	2.70%
No Response	9	9.10%	9	6.00%
Total	99	100.00%	149	100.00%
Android	86	86.90%	121	59.90%
Windows	6	6.10%	72	35.70%
iOS	2	2.00%	4	1.90%
No Response	5	5.00%	5	2.50%
Total	99	100.00%	202	100.00%

Regarding perceptions of content reliability, a significant proportion of students, 76 (76.8%), expressed a moderate perception of content reliability. However, satisfaction with internet services was relatively low, with only approximately one-third of the students, 33 (33.3%), indicating complete satisfaction (Table 5).

**Table 5:** Assessment of data reliability and internet service satisfaction (n=99).

	Good (Reliability on data)	Bad (Reliability on data)	Moderate (Reliability on data)	Satisfied completely	Not satisfied	Satisfied but not completely
Number (%)	14 (14.1%)	9 (9.1%)	76 (76.8%)	33 (33.3%)	16 (16.2%)	50 (50.5%)

Regarding the perceived benefits of internet use for study purposes, around 43 students (29.9%) found it advantageous due to the unlimited access to study materials. Additionally, 25 students (17.4%) acknowledged the availability of interesting study materials on the internet. A smaller percentage perceived the internet as providing easily accessible learning materials (15 students, 10.4%), saving time (10 students, 6.9%), and being cost-effective (8 students, 5.5%). Twenty-two students (15.3%) appreciated all the mentioned factors in favor of using the internet (Table 6).

**Table 6:** Perceived benefits of internet usage during studies across all participants (n=144).

Benefits	Number (Percentage)
Easily-assessed	15 (10.4%)
Cost-effective	8 (5.5%)
Unlimited access to study material	43 (29.9%)
Interesting	25 (17.4%)
Time-saving	10 (6.9%)
All of the above	22 (15.3%)
No response	21 (14.6%)

The most common challenging factor reported by internet users was slow internet speed, affecting 63 students (63.6%), followed by unavailability, affecting 11 students (11.1%) (Table 7).

**Table 7:** Distribution of study subjects according to the challenging factors in internet users.

Problems	Frequency of one major response used (most frequent)		Frequency (Multiple response)	
	Number	Percentage (%)	Number	Percentage (%)
Unavailability	11	11.10%	12	9.00%
Slow speed of internet	63	63.60%	89	66.90%
Wastage of time	8	8.10%	8	6.00%
Unreliable data	2	2.00%	8	6.00%
Costly asset	4	4.00%	5	3.80%
All of above	6	6.10%	6	4.50%
No Response	5	5.10%	5	3.80%
Total	99	100.00%	133	100.00%

## Discussion

The unprecedented global pandemic underscored the critical need for innovative educational tools, catapulting e-learning to the forefront as the primary mode of instruction during lockdown. Across various fields, including medical education, online classes have emerged as a widely adopted method for content delivery and student interaction. Despite the establishment of e-communication in teaching and learning during the early years of the new millennium, its acceptance as an adjunct tool has remained in its infancy, particularly in India. The significance of this study extends beyond exploring Internet usage trends among medical students; it offers valuable insights into the compliance issues that students face during online classes. The present generation of medical students, who are not initially trained in this manner, grapples with learning styles that may not optimally leverage technology. Furthermore, the role of teachers in imparting web-based education has yet to be fully incorporated into the curriculum, emphasizing the study's reflection on our preparedness for unexpected situations. Numerous studies conducted during the COVID-19 pandemic have shed light on the perception of online teaching methods. The influence of the Internet on the academic world, encompassing information, communication, education, and research, cannot be overstated. The primary objective of medical education is to promote the development of students' medical knowledge and instill commitment to continuous learning throughout their lives. Appropriate information retrieval skills and regular use of original scientific sources are critical components of this process [13]. In the current study, 75% of students preferred books for learning, and 37 out of 51 (72.5%) female participants used the internet for learning purposes. A study by Aldebasi YH and Ahmed MI showed that, in the context of information retrieval, 84% of males utilized the Internet, 36% relied on journals or libraries, and 35% used textbooks. Conversely, females showed a preference for textbooks (75%) and the Internet (14%) [14]. The present study revealed that 64 of the 99 (64.6%) participants who used the Internet for education came from urban areas. Loan FA found that 39.89% urban and 31.09% rural students use the internet for information and while 35.29% of rural students and only 23.50% of urban students use internet for education [15]. We found that Internet users for education were higher among public and convent-educated students than among government-educated students. The use of technology in classrooms with multiple ICT applications is likely to be responsible for these drivers. We also found that approximately 22 out of 31 (70.9%) average Hindi medium students used the Internet for educational purposes. Wikipedia (63.6%) and clinical keys (16.2%) were the most common digital learning tools used. The majority (59.6 percent) used mobile data to access the Internet. The challenges of unavailability, slow Internet use, waste of time, unreliable data, and slow Internet use have shown significant p-values in this study. Major obstacles of online learning mentioned by students were internet connectivity problems (79.8%), according to a cross-sectional study by Rao LP et al. [16]. Different studies on students’ Internet use have yielded various results. Sharma RR et al. found out that Undergraduate students used computers for entertainment and general information. Among postgraduate students, the tendency was to use computers generally for thesis and research purposes [17]. Inamdar SC and Rotti SB observed that 61% of the students used the Internet to watch movies and play games [18]. According to Challa N and Madras V, 60.55% used the Internet before joining a medical college, 14.45% used the Internet for university purposes, 29.45% used social media, and 56.1% used entertainment [19]. Taher E reported that 75% of Internet users accessed the web for irrelevant medical reasons [20]. A study found that 90% of students used e-mail on a regular basis and 80% used the Internet on a regular basis [21]. Kumar P found that 72.34% of respondents were comfortable and confident about using the Internet. Nearly 65.95% of the Internet users used the Internet to search for documentation, 63.82% used email, and 34.04% for used chat. Almost 55.31% of respondents had a shortage of PCs. A study done by Kumar P (2012) showed that 27.65% of students had no computer network facility on campus for their study program [22]. O'Carroll AM et al.'s study, students cited up-to-date resources, Google and Wikipedia, the most cited by medical students for their accessibility, understanding, and utility [23]. According to the Challa N and Madras V study, 93.89% of students use computers at home or at the hostel, and 36.11% use the Internet every day [19]. To obtain precise and reliable medical knowledge, it is imperative for students to possess the skills to effectively search for and retrieve objective, comprehensive, and trustworthy information [24]. Effective utilization of digital resources necessitates adequate infrastructure and Internet training. Students must develop skills to extract the necessary information from the Internet and websites. According to Mhlanga D's (2024) review article, formulating online education frameworks that fit the unique socioeconomic setting of developing countries depends on increased support for research and innovation, so promoting educational innovation and the creation of relevant content and pedagogical approaches [25]. This ability is crucial for evidence-based learning and enhances the quality of physician education. Hence, it is imperative for students to undergo training courses to acquire the skills of literature search and effective information extraction from the internet. It is advisable to offer brief training programs focused on computer applications and internet utilization in medical universities. Students should be educated in extracting relevant knowledge from websites. Furthermore, these abilities can enhance engagement in medical research.

Limitations

The research is constrained by its cross-sectional approach, which captures a momentary snapshot of internet use, thereby limiting the evaluation of long-term trends. The questionnaire was developed based on existing literature and underwent pretesting with a subset of participants to ensure clarity and relevance. In the questionnaire, 'Internet speed' was based on students' subjective perception. While formal validation measures (e.g., Cronbach's alpha) were not conducted, the instrument was refined based on pretesting feedback. The sample size was confined to first-year MBBS students from a single medical institution, potentially impacting the generalizability of the results to other areas or educational settings. The dependence on self-reported data may potentially lead to recall bias or mistakes in reporting internet use trends.

## Conclusions

The present study of internet usage trends among medical students underscores the changing digital educational environment. The findings highlight the necessity of customizing educational methodologies to properly utilize digital learning platforms, given the predominant reliance on Google Chrome and Wikipedia. Despite ongoing concerns like sluggish internet connections, the study underscores the necessity for a holistic strategy to tackle these problems, particularly with on-campus internet connectivity. The inadequate satisfaction with internet services indicates areas for enhancement that require further examination and action. Future research should investigate the efficacy of targeted interventions designed to improve content dependability and user satisfaction.

Furthermore, examining the influence of advancing technologies like artificial intelligence and virtual reality on medical education may yield significant insights into the future of digital learning. Comprehending the preferences and obstacles medical students encounter in the digital age is a continual endeavor. Longitudinal research examining shifts in internet usage habits and educational perspectives over time could enhance our understanding of the relationship between technology and medical education. As we explore the digital landscape, ongoing research is essential to guide educational policies and practices, ensuring that medical students possess the optimal tools and resources for success in their academic pursuits and future medical professions.

## Appendices

Questionnaire

Title: Exploring the Current Medical Learning: A Comprehensive Analysis of Internet-Usage Patterns and Educational Impact among Medical Students

Socio-economic Details:

**A.1** Student ID: ..........................

**A.2** Age (in years): ..........................

**A.3** Sex: 1. Male 2.Female

**A.4 **Professional Year: 1. 1st MBBS 2. 2nd MBBS 3. 3rd MBBS 4. 4th MBBS 5. BDS

**A.5** Background: 1. Rural 2. Semi-urban 3. Urban

**A.6** Schooling Type: 1. Government 2. Public 3. Convent 4. Missionary

**A.7** Medium of Education (before medical college): 1. English 2. Hindi 3. Others__________ (Please specify)

**A.8** Pre-Medical Education: 1. 12th 2. BSc3. MSc 4. Others_________________ (Please specify)

**A.9** Occupation of Parents: ......................................................

**A.10** Monthly Income of Parents: ......................................................

Section 1: Internet Accessibility and Devices

**B.1** Do you use the internet? 1. Yes 2. No

**B.2** How do you access the internet? (Select all that apply)

1. Personal device (Smartphone/Tablet/Laptop/Desktop)

2. Institutional Wi-Fi (Library/Computer Lab)

3. Cyber Café

4. Borrowing from friends

**B.3** What type of device do you predominantly use for internet access?

1. Smartphone

2. Tablet

3. Laptop

4. Desktop

**B.4** What is the operating system of your primary device?

1. Android

2. Windows

3. iOS

4. Others___________________ (Please specify)

**B.5** What is your primary source of internet access?

1. Institutional Wi-Fi

2. Mobile Data Pack

3. Dongle/Data Card

4. Broadband

5. Others_________________________ (Please specify)

Section 2: Internet Usage Patterns

**C.1** How did you learn to work with computers/internet? (Select all that apply)

1. Learning as a course subject in school

2. Self-learning (hit and trial)

3. Guided by friends

4. Observing others

**C.2** How frequently do you use the internet for academic purposes?

1. Daily

2. Weekly

3. Occasionally

**C.3** What activities do you use the internet for? (Select all that apply)

1. E-mailing, e-ticketing, e-booking, e-billing

2. Social media (WhatsApp/Facebook/Instagram)

3. Online courses (distant learning programs)

4. Accessing university websites (e-zone)

5. Video calling/chatting

6. Watching YouTube videos

7. Surfing/browsing

8. Watching news or TV serials

9. Downloading songs, films, or PDFs/Books

10. Online shopping

11. Playing online games

12. Preparing seminars, assignments, or PPTs

13. Studying difficult topics related to medical subjects

**C.4** Which search engine do you use most frequently?

1. Google Chrome

2. Safari/Bing/Yahoo/Firefox

3. Other (Please specify)

**C.5** Do you use any database for studying purposes? (Select all that apply)

1. ClinicalKey

2. PubMed

3. Google Scholar

4. Cochrane Library

5. Wikipedia

6. Others__________________________ (Please specify)

Section 3: Perceptions and Challenges

**D.1** How reliable do you find the content you access online?

1. Good

2. Moderate

3. Poor

**D.2** Do you always find satisfactory answers while studying using the internet?

1. Yes (always)

2. no (Never)

3. Sometimes

**D.3** Do you always rely on the information retrieved from the internet?

1. Yes (always)

2. no (Never)

3. Sometimes

**D.4** What challenges do you encounter while using the internet for learning? (Select all that apply)

1. Slow speed of the internet

2. Unavailability of internet

3. Distraction from non-academic activities

4. Unreliable data

5. Costly access

6. Others____________________ (Please specify)

Section 4: Benefits and Preferences

**E.1** What benefits do you perceive from using the internet for educational purposes? (Select all that apply)

1. Easily accessible

2. Cost-effective

3. Unlimited study material

4. Interesting way of learning

5. Time-saving

6. All of the above

**E.1** Which learning resource do you prefer?

1. Books

2. Internet

3. Both (Books and Internet)

Thank you for your participation!
